# Examining social determinants of health: the role of education, household arrangements and country groups by gender

**DOI:** 10.1186/s12889-019-7054-0

**Published:** 2019-06-06

**Authors:** Jordi Gumà, Aïda Solé-Auró, Bruno Arpino

**Affiliations:** 1 Department of Political and Social Sciences, Universitat Pompeu Fabra (UPF), Carrer Ramón Trias Fargas 25-27, 08005 Barcelona, Spain; 2 Sociodemography Research group (DemoSoc), University Pompeu Fabra (UPF), Barcelona, Spain; 3 Research and Expertise Centre for Survey Methodology (RECSM), Barcelona, Spain; 4 Department of Statistics, Computer Science, Applications, University of Florence, Florence, Italy

**Keywords:** Social determinants of health, Education, Household arrangements, Gender differences, Europe

## Abstract

**Background:**

The majority of empirical studies focus on a single Social Determinant of Health (SDH) when analysing health inequalities. We go beyond this by exploring how the combination of education (micro level) and household arrangements (mezzo level) is associated with self-perceived health.

**Methods:**

Our data source is the 2014 cross-sectional data from the European Survey of Living Conditions (EU-SILC). We calculate the predicted probabilities of poor self-perceived health for the middle-aged European population (30–59 years) as a function of the combination of the two SDHs. This is done separately for five European country groups (dual-earner; liberal; general family support; familistic; and post-socialist transition) and gender.

**Results:**

We observe a double health gradient in all the country groups: first, there is a common health gradient by education (the higher the education, the lower the probability of poor health); second, household arrangements define a health gradient within each educational level according to whether or not the individual lives with a partner (living with a partner is associated with a lower probability of poor health). We observe some specificity in this general pattern. Familistic and post-socialist transition countries display large differences in the predicted probabilities according to education and household arrangements when compared with the other three country groups. Familistic and post-socialist transition countries also show the largest gender differences.

**Conclusions:**

Health differences in European populations seem to be defined, first, by education and, second, by living or not living with a partner. Additionally, different social contexts (gender inequalities, educational profile, etc.) in European countries change the influences on health of both the SDHs for both women and men.

**Electronic supplementary material:**

The online version of this article (10.1186/s12889-019-7054-0) contains supplementary material, which is available to authorized users.

## Background

Studies on the social determinants of health (SDH) have contributed to a better understanding of health inequalities within and across populations, and have given important support to the design of public health policies [26, 27]. As Dahlgren and Whitehead [10] proposed in their ‘Rainbow Model’, the social root of SDHs implies that they can be classified according to the social context to which they belong (from individual characteristics to the general context that is common for a large population). According to the Rainbow Model, all SDHs identified by the literature can be classified into three levels, according to whether they correspond to individual (micro level) or contextual characteristics (mezzo and macro levels). For the latter, we should distinguish between SDHs pertaining to the mezzo level of the closest context (e.g. household and family, place of residence, etc.) and macro level factors of the most general context (e.g. public policies, sociocultural characteristics, etc.).

An exhaustive review of the literature demonstrates that the majority of empirical studies focus on a single SDH, which has contributed to having a detailed knowledge about how each single factor individually influences health inequalities. Among these SDHs, most attention has been given to socioeconomic characteristics of individuals (education, activity status, salary, etc.) [1]. However, contextual level factors such as household arrangements (mezzo level) [22] and public health policies (macro level) [31] have also shown a high capacity to explain health differences. In other words, a picture defined merely by individual features cannot fully capture the complexity of modern societies when we attempt to explain health differences. The only exception to this is a recent case study for the Spanish adult population by Gumà et al. [18] where the authors stated that combining information from education and household arrangements permits the definition of more precise health profiles.

Our aim is to go beyond the study of a single SDH by exploring the interactions between SDHs at different levels in order to assess whether possible advantages or disadvantages related to an individual’s context are modified depending on their individual features, and vice versa. For this purpose we examine the combination between educational level (as a proxy of long-term social differences beyond contextual factors like employment status or salary) and household arrangements (the most basic unit of socialization between relatives), two outstanding SDHs from the micro and mezzo levels, among the middle-aged European population (30–59 years). Furthermore, we account for the most general context by adopting a comparative perspective and analysing how the effects of the above mentioned SDHs vary across different European regions according to the type of family welfare regime in those regions [34]. Welfare regimes permit us to summarize the general context (e.g. public policies, levels of gender equity, etc.) of European countries in some way.

The complexity of the interplay between the SDHs under consideration is even greater when we consider gender inequalities. It has been proved that gender inequalities in western countries lead to different signs and magnitudes of the effect of a particular SDH on females’ and males’ health [39] (e.g. employment status shows a stronger association with male health, whereas educational attainment is more relevant for female health).

To the best of our knowledge, no previous study has examined the influence on health of the combination between education and household arrangements, although in some cases the relationship between household arrangements and health has been explored with education being included as a control variable [23, 29]. We hypothesize that the household effect on health is moderated by education because of its ability to counteract possible negative situations [20] (e.g. the social network of an individual with higher education has been shown to be of help in finding a new job during an episode of unemployment). Additionally, although household arrangements have displayed a higher explanatory capacity of health variability among women [29] than among men, we expect to find lower gender differences in health according to household arrangements in regions with higher gender equality. Finally, we also assume that differences between European country groups in terms of family, educational and gender inequality profiles may moderate the effects of the SDH variables at lower levels.

### Education and health

The association between health and education has been repeatedly tested because of the capacity of the latter to establish different levels of social stratification [20]. Population groups defined by a low educational level show a greater disadvantage in terms of health, although there are differences between countries according to their specificities regarding health behaviours and public policies [6, 20, 28]. Individuals with the lowest educational level have been consistently found to report the worse health [35]. Indeed, education has been shown to influence an individual’s health at different life-course stages (from adulthood to advanced age), as well as to mediate the long-term influence of early-life conditions on health [3].

Educational differences in health across Europe are well documented, with a general pattern of large variations in the magnitude of the differentials across countries. In general, a high level of social transfer is expected to reduce exposure to deprivation, and this could be translated into reductions in the health disadvantage of poorly educated groups. For instance, for the Spanish population with a low educational level, Alcañiz et al. [2] found a higher prevalence of certain lifestyle indicators such as tobacco and alcohol consumption and a sedentary lifestyle, in addition to greater problems in performing daily activities. Additionally, the magnitude of the influence of education on health differs between women and men. According to the resource substitution theory, the absence of one or more socio-economic resources can be replaced by a greater influence from other resources [36]. As a consequence, lower female participation in the labour market, as well as the gender wage gap, has reinforced the importance of education for health among women [36, 37].

### Household arrangements and health

Household arrangements, as an SDH, is located at the intermediate level of the Rainbow Model. Household arrangements represent the context in which individuals with a family tie perform a daily exchange of resources of diverse natures (economic, emotional, care, information, etc.) [39]. Focusing on specific family ties, those living in a couple have been found to report better health than their counterparts who are living without a partner [23]. This evidence has received several explanations: 1) higher levels of social control might reduce the propensity to carry out risky behaviour, which is especially beneficial for men; 2) there may be an optimization of resources through scale economies; and 3) the creation and maintenance of a larger social network can be of help in adversity [16, 41].

Living with children is another relevant family tie that has shown both positive and negative effects on health. The positive effects, like the increase in life satisfaction due to emotional reward, are explained because of feelings of fulfilling a vital purpose [4, 19]. However, detrimental consequences on well-being and health have also been found, due to changes in the economic capacity of the household, increases in couple conflicts or difficulties in balancing the family and work spheres, especially for women [25, 30].

The relationship between household arrangements and health has also been found to vary among countries. A recent study [13] that assessed the association between household arrangements and self-perceived health among the adult population in 12 European countries found that the usual health gap between partnered and non-partnered subpopulations is smaller in countries where the relative importance of the second group is higher. The authors also pointed to a meaningful different explanatory capacity of household arrangements on the health variability of women and men, with this being an SDH that is more relevant for females.

## Methods

We used the 2014 cross-sectional data from the European Survey of Living Conditions (EU-SILC). This survey takes the household as a sampling unit and collects information for each member of the household, except in seven countries (Denmark, Finland, Iceland, the Netherlands, Norway, Sweden and Slovenia) where only one member of the household was randomly selected to answer the entire questionnaire. As a result of the influence of age on family events we restricted our sample to individuals aged 30 to 59: for instance, in Spain, Italy, Portugal, Croatia, Greece and Bulgaria, among others, the average age on leaving the parental home was about 28 and 30 for women and men, respectively, in 2013 [15]. Moreover, we tried to avoid possible bias from the association between health and age of retirement across countries [12]. For instance, the lowest effective retirement age for men was found in France (59.4), while the lowest age for women was found in Slovakia (58.2) [32]. Respondents born in a different country, and those who stated that they were unable to work because of their health, were not included in our analysis. Cyprus was also not included because of its political specificities. After dropping 3% of observations with missing cases from the original sample, which were randomly distributed according to country, age and gender, the final sample consisted of 187,898 respondents (52% women and 48% men).

Adopting the measure proposed by the WHO [11], our dependent variable was self-perceived health, which was measured with the question ‘How is your health in general?’. This is one of the three health questions that pertain to the Minimum European Health Module whose reliability and comparability between European countries has been previously confirmed [8]. This indicator was chosen on the basis of its proven capacity to give information about a person’s general current health status as well as about any recent changes [21]. Self-perceived health is especially suitable for studying middle-aged populations where morbidity levels are still low but future health problems are incipient. Indeed, self-perceived health has showed a stronger association with mortality, an outcome of objective health, at younger ages [5, 17]. Following common practice [9], we grouped the five possible answers into two categories: good or very good health (good health = 0), and fair, bad or very bad health (poor health = 1).

Education was grouped into three categories: low (primary – whether or not complete – and low secondary studies), medium (upper secondary and post-secondary but non-tertiary education) and high (tertiary). Household arrangements were defined according to whether or not the individual lived with a partner and/or with children, resulting in four different categories: 1) living with neither partner nor children (single-person household or living with other people); 2) living with partner but without children; 3) living with partner and children; and 4) living with children but without partner (single parent).

To explore how the combination between education and household arrangements was associated with self-perceived health, we combined these and created a new variable with 12 categories. We opted for the combination of both variables after testing the significance of the interaction of both variables, both overall and by gender (Additional file 1: Table S1 and Additional file 2: Table S2). We also tested the triple interaction between education, household arrangement and gender (Additional file 3: Table S3).

Following Oláh et al. [34], we grouped the 30 countries in the study into five groups according to the type of family welfare regime: dual-earner (Denmark, Finland, Iceland, Norway and Sweden); liberal (Switzerland, United Kingdom, Ireland and Malta); general family support (Austria, Belgium, Germany, France and Netherlands); familistic (Greece, Spain, Italy and Portugal); and post-socialist transition (Bulgaria, Czech Republic, Estonia, Croatia, Hungary, Latvia, Lithuania, Poland, Romania, Serbia, Slovenia and Slovak Republic).

We ran separate logistic regressions by country groups and gender after assessing the significant difference between the estimates across the five European regions as well as between women and men in a pooled model (Additional file 4: Table S4). The reason for calculating independent models according to these two factors, gender and country groups, is twofold. First, separate models according to gender in addition to restricting individuals in our analysis aged 30–59 prevents from a possible issue of dependency in our analysis due to the inclusion of individuals from the same household. The age selection prevents from analysing members of the same family from two different generations and separate models by gender imply that members from a couple are in different models (same sex couples are rare in the EU-SILC data). Second, previous research has proved that the answer to the question about self-perception of health is sensitive to gender and cultural context [33].

All models included the combination of education and household arrangements in order to assess possible differences in the health gradient observed in previous research when both variables were analysed separately. In all models we controlled for socio-economic and demographic variables that had previously been proved to have an association with health: age, employment status (employed, unemployed and inactive) and subjective economic capacity of the household to make ends meet (easily, fairly easily, with some difficulty and with difficulties). To ease the interpretation of results, we present, separately for each country group and gender, the predicted probabilities of poor health with 95% confidence intervals from the logistic models including all control variables (complete estimates of all models are available in Additional file 5: Table S5).

## Results

Table 1 reports descriptive statistics of the educational and household arrangements profile by gender, revealing meaningful differences between the five country groups. Women display higher levels of educational attainment, with the highest gender difference in the dual-earner countries. In general, focusing on country groups differences we observe that dual-earner, liberal and general family support countries show higher percentages of high education than post-socialist and familistic countries, with the majority of the population in the post-socialist countries being concentrated at the medium educational level and in the familistic countries at the low education level.

**Table 1:** Educational attainment and household arrangements by European groups of countries and gender (ages 30–59) 2014

		Dual-earner countries		Liberal		General family support		Familistic		Transition post-socialist	
		Men (%)	Women (%)	Men (%)	Women (%)	Men (%)	Women (%)	Men (%)	Women (%)	Men (%)	Women (%)
Low education	No partner-no children	3.7	1.4	7.1	3.1	3.1	2.1	9.7	4.1	3.8	1.3
	No partner-children	0.3	1.3	0.9	4.9	0.3	1.9	0.8	4.4	0.4	2.1
	Partner-no children	3.4	3.5	4.2	4.6	2.8	3.9	5.5	6.7	1.9	2.9
	Partner-children	5.0	3.4	15.4	14.5	5.4	5.5	24.2	22.7	6.7	7.9
	Total	12.3	9.7	27.6	27.1	11.5	13.3	40.2	37.9	12.8	14.2
Medium education	No partner-no children	9.6	4.7	7.1	4.4	10.5	7.0	10.1	5.4	14.7	6.6
	No partner-children	1.8	4.0	0.9	4.8	1.3	5.8	0.5	3.5	1.5	7.8
	Partner-no children	10.8	10.1	6.8	7.3	11.7	12.1	4.8	4.8	11.2	10.8
	Partner-children	24.7	17.4	18.1	20.5	25.3	25.1	20.5	20.3	40.7	33.6
	Total	46.9	36.2	32.9	37.1	48.8	50.0	35.9	34.0	68.1	58.8
High education	No partner-no children	6.3	6.5	6.7	5.7	7.2	6.2	6.9	6.3	4.0	4.4
	No partner-children	1.2	4.8	0.6	3.5	1.1	3.7	0.3	2.3	0.3	3.4
	Partner-no children	8.9	11.3	9.1	7.1	8.7	7.3	3.4	4.1	3.4	4.2
	Partner-children	24.3	31.4	23.2	19.6	22.6	19.7	13.4	15.4	11.4	15.0
	Total	40.7	54.1	39.6	35.9	39.6	36.8	23.9	28.1	19.1	27.0
Total		100.0	100.0	100.0	100.0	100.0	100.0	100.0	100.0	100.0	100.0
	N	7156	7054	9881	11,423	16,140	17,718	21,600	23,145	34,310	39,471

Data source: EU-SILC 2014

Note: Dual-earner (Denmark, Finland, Island, Norway and Sweden); Liberal (Switzerland, United Kingdom, Ireland and Malta); General family support (Austria, Belgium, Germany, France and Netherlands); Familistic (Greece, Spain, Italy and Portugal); and Transition post-socialist (Bulgaria, Czech Republic, Estonia, Croatia, Hungary, Latvia, Lithuania, Poland, Romania, Serbia, Slovenia and Slovak Republic

As for household arrangements, the two situations of living with a partner (with or without children) are the most frequent, with the only exception being the familistic countries, where the category of those living without a partner or children ranks second (except for poorly educated women). Living without children is the most frequent situation among those who do not live with a partner, whereas in general the least frequent is living with children but no partner. Overall, gender differences are similar within each country group. The most meaningful difference relates to the higher percentage of single mothers compared to single fathers, whereas men show a higher prevalence of living with neither partner nor children.

The prevalence of poor health according to education, household arrangements and gender by country groups (Table 2) reveals a double health gradient according to the combination of education and household arrangements: the higher the educational attainment, the lower the prevalence of poor health; and, within each educational level, those who live with a partner declare better health outcomes, which become even better when they also live with children. However, it seems that the educational gradient prevails even for the same categories of household arrangements: those who have a certain household arrangement show better or worse health status depending on their educational status.

**Table 2:** Prevalence of poor health according to educational attainment and household arrangements by European groups of countries and gender (ages 30–59) 2014

		Dual-earner countries		Liberal		General family support		Familistic		Transition post-socialist	
		Men (%)	Women (%)	Men (%)	Women (%)	Men (%)	Women (%)	Men (%)	Women (%)	Men (%)	Women (%)
Low education	No partner-no children	34.1	44.1	25.3	36.3	37.6	48.9	29.1	43.3	42.2	62.1
	No partner-children	22.7	30.9	27.1	31.1	29.8	44.9	31.8	43.1	46.7	55.8
	Partner-no children	30.4	33.2	21.5	31.5	30.9	41.5	36.9	45.1	46.5	56.8
	Partner-children	22.8	24.6	17.6	21.6	27.2	32.5	28.0	34.1	36.1	44.5
Medium education	No partner-no children	26.9	28.7	20.0	22.0	28.4	30.2	13.1	18.7	28.2	39.8
	No partner-children	22.1	23.6	17.6	22.0	24.0	31.6	16.5	24.1	34.9	39.4
	Partner-no children	20.7	24.2	19.1	18.6	27.8	29.4	16.6	23.2	40.3	45.5
	Partner-children	17.1	17.0	14.2	15.1	21.4	20.2	13.9	15.8	27.4	28.8
High education	No partner-no children	18.9	17.6	13.9	15.9	17.5	20.3	8.8	14.2	15.7	22.2
	No partner-children	12.5	16.4	18.6	15.3	14.6	20.0	11.5	18.7	20.8	26.7
	Partner-no children	10.7	14.3	10.0	11.0	15.1	18.0	10.3	13.7	24.1	28.6
	Partner-children	9.0	10.7	10.0	9.8	12.5	13.1	10.4	10.4	15.4	17.0
	Total	17.4	17.7	15.4	17.8	21.1	23.7	19.3	24.1	28.7	33.1

Data source: EU-SILC 2014

Note: Dual-earner (Denmark, Finland, Island, Norway and Sweden); Liberal (Switzerland, United Kingdom, Ireland and Malta); General family support (Austria, Belgium, Germany, France and Netherlands); Familistic (Greece, Spain, Italy and Portugal); and Transition post-socialist (Bulgaria, Czech Republic, Estonia, Croatia, Hungary, Latvia, Lithuania, Poland, Romania, Serbia, Slovenia and Slovak Republic

In general, we observe a general health advantage of men compared to women [7, 40]. A country group gradient is also evident, with better health outcomes in dual-earner and liberal countries, followed by familistic, general family support and post-socialist transition countries, which lie in the region with the worst health outcomes.

**Fig. 1:**
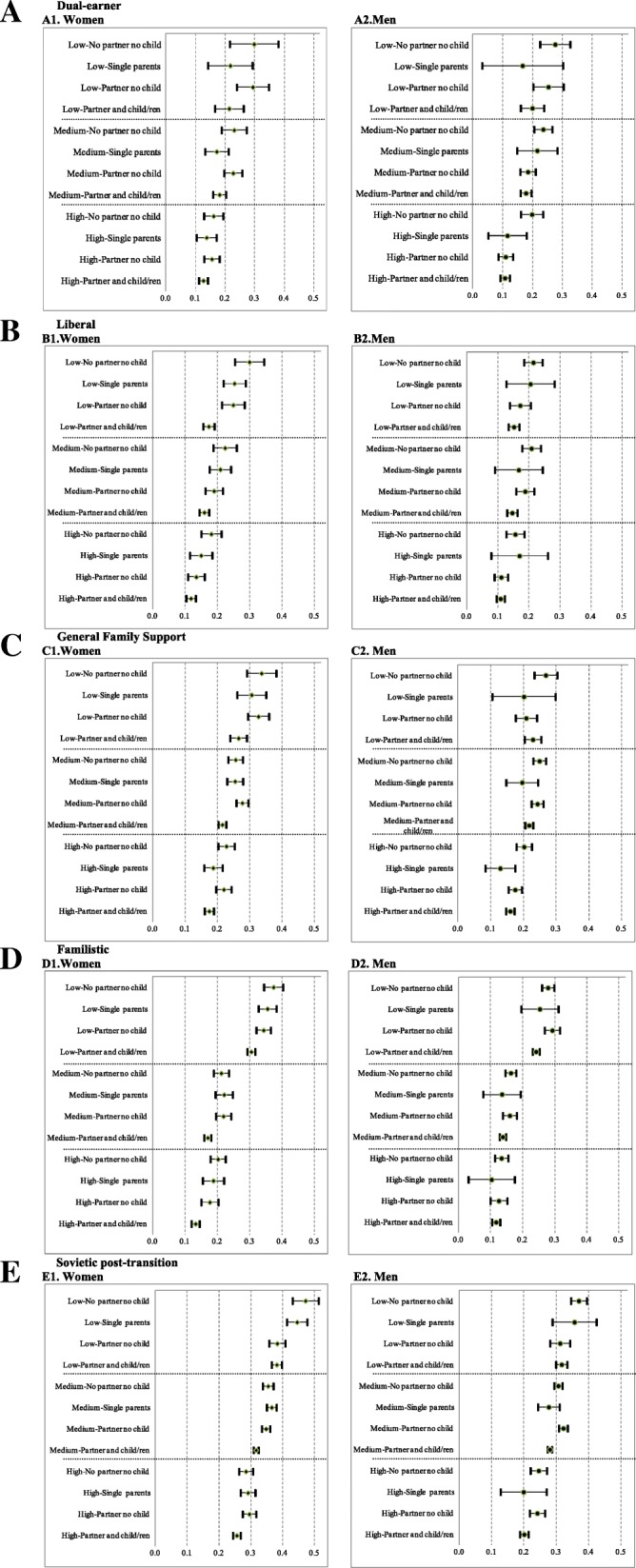
Predicted probability of declaring poor health with 95% confidence intervals as function of combining educational attainment and household arrangement by gender and European groups of countries. 2014. **A** Dual-earner. A1. Women. A2.Men. **B** Liberal. B1.Women. B2.Men. **C** General Family Support. C1.Women. C2. Men. **D** Familistic. D1.Women. D2. Men. **E** Sovietic post-transition. E1. Women. E2. Men. Panel **A** for Dual-earner countries, panel **B** for Liberal countries, panel **C** for General Family Support, panel **D** for Familistic countries, and panel **E** for Sovietic post-transition countries. Sub-panel 1 for Women and Sub-panel 2 for Men. Data source: EU-SILC 2014. Note1: Low (Low educational attainment); Medium (Medium educational attainment); High (High educational attainment). Note2: Dual-earner (Denmark, Finland, Island, Norway and Sweden); Liberal (Switzerland, United Kingdom, Ireland and Malta); General family support (Austria, Belgium, Germany, France and Netherlands); Familistic (Greece, Spain, Italy and Portugal); and Transition post-socialist (Bulgaria, Czech Republic, Estonia, Croatia, Hungary, Latvia, Lithuania, Poland, Romania, Serbia, Slovenia and Slovak Republic

The probabilities of poor health predicted from the logistic models (Fig. 1) confirm the double gradient observed when combining educational attainment and household arrangements. First, there is a common educational gradient in all the country groups, so that the higher the education, the lower the probability of declaring poor health. Second, there is a health gradient within each educational level according to household arrangements, mainly defined by whether or not an individual lives with a partner (living with a partner is associated with a lower probability of poor health), whereas having children only shows a small effect when education is taken into account. It must be noted that in the specific case of single mothers (the low number of single fathers does not permit us to draw conclusions for them), which is the situation that the literature indicates as the most disadvantaged in terms of health [38], a significantly lower probability of poor health is observed when the educational status is higher, in all the country groups except dual-earner countries.

Although this is a general pattern regardless of gender and country groups, we also observe specificities regarding the magnitude of the probabilities in each country group. In general terms, the highest probabilities of having poor health are observed in the post-socialist cluster, whereas the liberal and dual-earner countries display the lowest values. When we focus on the combination of education and household arrangements, familistic and post-socialist transition countries display large differences in the predicted probabilities when compared with dual-earner, liberal and general family support countries. Familistic countries show the widest gap between the lowest educational level and the other two levels, whereas in the post-socialist transition countries the most noticeable difference is observed between the highest educational level and the level of low and medium education. On the other hand, the other three country groups (dual-earner, liberal and general family support) follow the general pattern described above, with progressive differences in the probability of poor health according to educational status.

By gender, the probability of poor health is higher for women in general, with the largest gender difference being found in the familistic and post-socialist transition countries, whereas general family support, dual-earner and liberal countries show the lowest differences. More specifically, the most striking gender differences are found among poorly educated individuals in familistic and post-socialist transition countries.

## Discussion

This study explores the differences in self-perceived health among middle-aged (30–59) Europeans, by combining information on educational attainment and household arrangements, two well-studied SDHs from the micro and the mezzo levels that have been considered separately in previous studies. We show different specificities according to gender and groups of European countries (dual-earner, liberal, general family support, familistic and post-socialist transition).

Our results display a double health gradient defined according to the combination of education and household arrangements. Specifically, at the micro level the educational health gradient prevails (the higher the educational status, the better the health outcomes), but we also observe an additional health gradient within each educational level according to the type of household arrangement. This health gradient is located at the mezzo level and seems to be mainly defined by whether or not the individual lives with a partner, whereas living with children does not seem to be relevant when education is controlled for. When taking into account both SDHs together, we see that not only do individuals declare better or worse health outcomes within the same educational level depending on their household arrangement, but also that health differences between educational levels depend on the type of household arrangement. The case of single mothers stands out (there are too few single fathers to draw conclusions), showing the highest probabilities of poor health among poorly educated individuals (together with single childless people), whereas their probabilities are not significantly different from those of people in other household arrangements among highly educated individuals. The fact that single mothers do not display significant differences regarding the other household arrangements within the same educational level points to the fact that previous results on health differences by household arrangements were importantly moderated by education.

The separate models by country groups contribute to the discovery of certain specificities within the general pattern in the association between the two SDHs and self-perceived health. The most outstanding health difference is the sharp gap between those with low educational attainment and the rest of the population in the familistic countries, and between those with high education and the rest of the population in the post-socialist transition countries. Additionally, these two country groups show the largest gender differences in the health gradient according to education and household arrangements. Although men usually show better health outcomes than women [33], dual-earner, liberal and general family support countries display the lowest gender difference, whereas familistic and post-socialist transition countries show the highest. Indeed, in the first three country groups there is almost no gender difference within the same combination of education and household arrangements, whereas this is not the case in the last two groups of countries. Therefore, the worse aggregated health profile in these countries [14] seems to be basically defined by their specific educational profile as well as by their lower level of gender equality [24].

Overall, the main contribution of this paper is twofold. First, we have shown that combining information from two SDHs, representing the micro and the mezzo levels, leads to more accurate insights into the most vulnerable socio-demographic profiles in terms of health. Second, although both SDHs contribute towards explaining health differences among European populations, education (micro level) seems to explain a greater amount of health variability than whether or not an individual lives with a partner (mezzo level). Additionally, we have uncovered meaningful gender differences in the association between education, household arrangements and health in the five country groups, pointing out that current gender inequalities in western societies mean that the influence of SDHs on health for women and men is different.

This study has also some limitations. First, the cross-sectional nature of our data does not allow to go further than only stating associations between variables. This does not permit us to explore possible mechanisms like selection into marriage and fertility due to different levels of educational attainment. Longitudinal data would also allow to compare the results from different generations in order to assess whether the association between our variables of interest and self-perceived health varies over time. Second, separate models according to country clusters only confirm the existence of contextual differences but do not permit to identify their origin. For this reason, we plan to incorporate information about SDHs from the macro level (e.g., public health expenditure in each country, general levels of gender equity, etc.) in future research in order to better understand how these factors interplay with SDHs from the micro and the mezzo levels to establish health differences.

## Conclusion

To conclude, this study contributes to confirm the idea that SDHs are interrelated, as it was already pointed out in a similar case study using only data for Spain [18], and that the analysis of their interactions could complement the current knowledge that we have about their separate influences on health.

## Additional files

Additional file 1: **Table S1** Odds ratio of poor self-perceived health of the interaction between education and household arrangements from the pooled logistic regression model for middle-aged Europeans (30–59 years old). This file confirms the statistical significance of the interaction between education and household arrangements for the whole working sample. (DOCX 15 kb)

Additional file 2: **Table S2** Odds ratio of poor self-perceived health of the interaction between education and household arrangements from the pooled logistic regression model for middle-aged Europeans (30–59 years old) by gender. This file contains the results from the model that confirms the statistical significance of the interaction between education and household arrangements for women and men separately. (DOCX 17 kb)

Additional file 3: **Table S3** Odds ratio of poor self-perceived health for the interaction between education, household arrangements and gender from the pooled logistic regression model for middle-aged Europeans (30–59 years old). This file shows the results from the triple interaction between education, household arrangement and gender. (DOCX 15 kb)

Additional file 4: **Table S4** Odds ratio of poor self-perceived health from the pooled logistic regression model for middle-aged Europeans (30–59 years old). This file confirms the significant difference between the estimates across the five European regions as well as between women and men. (DOCX 15 kb)

Additional file 5: **Table S5** Odds ratio of poor self-perceived health for middle-aged population (30–59 years old) by country cluster and gender This file contains the complete estimates of all models included in the Results section. (DOCX 16 kb)

## Data Availability

The data that support the findings of this study are available from third parties: for EU-SILC this is Eurostat (http://ec.europa.eu/eurostat/web/microdata/european-union-statistics-on-income-and-living-conditions).

## Abbreviations

EU-SILC: European Survey of Living Conditions
SDH: Social determinants of health
