# Supplementary material for: Examining social determinants of health: the role of education, household arrangements and country groups by gender
Source: BMC Public Health. 2019 Jun 6;19:699. doi: 10.1186/s12889-019-7054-0 (PMC6555096; doi:10.1186/s12889-019-7054-0)
Supplement: Supplementary file 1 — Table S1 Odds ratio of poor self-perceived health of the interaction between education and household arrangements from the pooled logistic regression model for middle-aged Europeans (30–59 years old). This file confirms the statistical significance of the interaction between education and household arrangements for the whole working sample. (DOCX 15 kb) [file 12889_2019_7054_MOESM1_ESM.docx]

**S.1. Odds ratio of poor self-perceived health of the interaction between education and household arrangements from the pooled logistic regression model for middle-aged Europeans (30-59 years old)**

Controlled for: Gender, Employment status, Household capacity to make ends meet, Country clusters and Age

Note: † p < 0.10; * p < 0.05; ** p < 0.01; *** p < 0.001.
