# Supplementary material for: Examining social determinants of health: the role of education, household arrangements and country groups by gender
Source: BMC Public Health. 2019 Jun 6;19:699. doi: 10.1186/s12889-019-7054-0 (PMC6555096; doi:10.1186/s12889-019-7054-0)
Supplement: Supplementary file 2 — Table S2 Odds ratio of poor self-perceived health of the interaction between education and household arrangements from the pooled logistic regression model for middle-aged Europeans (30–59 years old) by gender. This file contains the results from the model that confirms the statistical significance of the interaction between education and household arrangements for women and men separately. (DOCX 17 kb) [file 12889_2019_7054_MOESM2_ESM.docx]

**S.2. Odds ratio of poor self-perceived health of the interaction between education and household arrangements from the pooled logistic regression model for middle-aged Europeans (30-59 years old) by gender.**

Controlled for: Employment status, Household capacity to make ends meet, Country clusters and Age

Note: † p < 0.10; * p < 0.05; ** p < 0.01; *** p < 0.001.
