# Supplementary material for: Examining social determinants of health: the role of education, household arrangements and country groups by gender
Source: BMC Public Health. 2019 Jun 6;19:699. doi: 10.1186/s12889-019-7054-0 (PMC6555096; doi:10.1186/s12889-019-7054-0)
Supplement: Supplementary file 3 — Table S3 Odds ratio of poor self-perceived health for the interaction between education, household arrangements and gender from the pooled logistic regression model for middle-aged Europeans (30–59 years old). This file shows the results from the triple interaction between education, household arrangement and gender. (DOCX 15 kb) [file 12889_2019_7054_MOESM3_ESM.docx]

**S.3. Odds ratio of poor self-perceived health for the interaction between education, household arrangements and gender from the pooled logistic regression model for middle-aged Europeans (30-59 years old).**

Controlled for: Employment status, Household capacity to make ends meet, Country clusters and Age

Note: † p < 0.10; * p < 0.05; ** p < 0.01; *** p < 0.001.
