# Supplementary material for: Examining social determinants of health: the role of education, household arrangements and country groups by gender
Source: BMC Public Health. 2019 Jun 6;19:699. doi: 10.1186/s12889-019-7054-0 (PMC6555096; doi:10.1186/s12889-019-7054-0)
Supplement: Supplementary file 4 — Table S4 Odds ratio of poor self-perceived health from the pooled logistic regression model for middle-aged Europeans (30–59 years old). This file confirms the significant difference between the estimates across the five European regions as well as between women and men. (DOCX 15 kb) [file 12889_2019_7054_MOESM4_ESM.docx]

**S.4. Odds ratio of poor self-perceived health from the pooled logistic regression model for middle-aged Europeans (30-59 years old)**

Controlled for: Employment status, Household capacity to make ends meet and Age

Note: † p < 0.10; * p < 0.05; ** p < 0.01; *** p < 0.001.

Note: Dual-earner (Denmark, Finland, Island, Norway and Sweden); Liberal (Switzerland, United Kingdom, Ireland and Malta); General family support (Austria, Belgium, Germany, France and Netherlands); Familialistic (Greece, Spain, Italy and Portugal); and Transition post-socialist (Bulgaria, Czech Republic, Estonia, Croatia, Hungary, Latvia, Lithuania, Poland, Romania, Serbia, Slovenia and Slovak Republic
