# Supplementary material for: Examining social determinants of health: the role of education, household arrangements and country groups by gender
Source: BMC Public Health. 2019 Jun 6;19:699. doi: 10.1186/s12889-019-7054-0 (PMC6555096; doi:10.1186/s12889-019-7054-0)
Supplement: Supplementary file 5 — Table S5 Odds ratio of poor self-perceived health for middle-aged population (30–59 years old) by country cluster and gender This file contains the complete estimates of all models included in the Results section. (DOCX 16 kb) [file 12889_2019_7054_MOESM5_ESM.docx]

**S.5.Odds ratio of poor self-perceived health for middle-aged population (30-59 years old) by country cluster and gender**

Controlled for: Employment status, Household capacity to make ends meet and Age

Note: † p < 0.10; * p < 0.05; ** p < 0.01; *** p < 0.001.

Note: Dual-earner (Denmark, Finland, Island, Norway and Sweden); Liberal (Switzerland, United Kingdom, Ireland and Malta); General family support (Austria, Belgium, Germany, France and Netherlands); Familialistic (Greece, Spain, Italy and Portugal); and Transition post-socialist (Bulgaria, Czech Republic, Estonia, Croatia, Hungary, Latvia, Lithuania, Poland, Romania, Serbia, Slovenia and Slovak Republic
